# Gadolinium Tagged Osteoprotegerin-Mimicking Peptide: A Novel Magnetic Resonance Imaging Biospecific Contrast Agent for the Inhibition of Osteoclastogenesis and Osteoclast Activity

**DOI:** 10.3390/nano8060399

**Published:** 2018-06-02

**Authors:** Lubinda Mbundi, Steve T. Meikle, Rosa Busquets, Nicholas G. Dowell, Mara Cercignani, Matteo Santin

**Affiliations:** 1 Department of Surgical Research, Northwick Park Institute for Medical Research, University College London (UCL), Northwick Park & St Marks Hospitals, Watford Road, Harrow, Middlesex HA1 3UJ, UK; l.mbundi@ucl.ac.uk; 2 Centre for Regenerative Medicine and Devices, School of Pharmacy and Biomolecular Sciences, University of Brighton, Huxley Building, Lewes Road, Brighton BN2 4GJ, UK; stevenmeikle@googlemail.com; 3 Faculty of Science, Engineering and Computing, Penrhyn Road, Kingston University, Kingston Upon Thames KT1 2EE, UK; r.busquets@kingston.ac.uk; 4 Clinical Imaging and Science Centre (CISC), Centre for Regenerative Medicine and Devices, Brighton and Sussex Medical School, Lewes Road, Brighton BN1 9RR, UK; N.G.Dowell@bsms.ac.uk (N.G.D.); M.Cercignani@bsms.ac.uk (M.C.)

**Keywords:** osteoclastogenesis, RANK-RANKL-OPG, mimetic peptide, Gadolinium chelate, MRI

## Abstract

The control of osteoblast/osteoclast cross-talk is crucial in the bone remodelling process and provides a target mechanism in the development of drugs for bone metabolic diseases. Osteoprotegerin is a key molecule in this biosignalling pathway as it inhibits osteoclastogenesis and osteoclast activation to prevent run-away bone resorption. This work reports the synthesis of a known osteoprotegerin peptide analogue, YCEIEFCYLIR (OP3-4), and its tagging with a gadolinium chelate, a standard contrast agent for magnetic resonance imaging. The resulting contrast agent allows the simultaneous imaging and treatment of metabolic bone diseases. The gadolinium-tagged peptide was successfully synthesised, showing unaltered magnetic resonance imaging contrast agent properties, a lack of cytotoxicity, and dose-dependent inhibition of osteoclastogenesis in vitro. These findings pave the way toward the development of biospecific and bioactive contrast agents for the early diagnosis, treatment, and follow up of metabolic bone diseases such as osteoporosis and osteosarcoma.

## 1. Introduction

The physiological remodelling of bone tissue is controlled by a delicate balance between the activity of the cells producing its mineralised extracellular matrix, the osteoblasts, and that of the osteoclasts, the cells responsible for its resorption [1,2]. This process of tissue turnover is regulated through the interaction of the receptor activator of nuclear factor kappa-B (RANK), expressed by osteoclast progenitors, with its cognate ligand (RANKL), which is expressed by the osteoblasts either as a membrane-bound form or as a free-soluble form. The soluble form activates both osteoclast differentiation and activation, thus initiating bone resorption [2]. A fine regulation of the cross-talk between these two cells is provided by the synthesis of another protein, osteoprotegerin (OPG), a soluble receptor for RANKL that is secreted by osteoblasts and bone marrow stromal cells to decoy the recognition of RANK by RANKL [3]. By binding RANKL, OPG prevents the ligand recognition of RANK, thus inhibiting osteoclast differentiation and activity and allowing new bone tissue deposition [4].

Indeed, the balanced sequence of interactions of the RANK/RANKL/OPG triad represents a tightly controlled system at the centre of the osteoclast-osteoblast functional unit that determines skeletal mass at any time in an organism’s life. It is well established that the dysregulation of the RANK/RANKL/OPG triad significantly affects osteoclast maturation, survival, and activity, resulting in disorders of the bone remodelling cycle such as osteoporosis, Paget’s disease, rheumatoid arthritis, and bone metastases [5,6]. Current pharmacological interventions are designed to either reduce bone resorption or stimulate bone formation. Drugs used to reduce bone resorption include hormone replacement therapy (HRT) and selective oestrogen receptor modulators (SERM), bisphosphonates and those used to increase bone formation include teriparatide (PTH 1-34), and strontium renalate [7,8]. However, these drugs have associated side effects such as hot flashes, osteonecrosis, and poor fracture healing and osteointegration [8]. Moreover, as RANKL plays other physiological roles including immune function, lymph node formation, and mammalian development [9], the development of therapeutic drugs that can replace its use with no side effects is desirable. One of the notable developments in this regard is the recently approved drug, Denosumab, a fully human monoclonal anti-RANK antibody that mimics the activities of OPG, which is used as a bone anti-resorptive agent [10,11]. However, this drug is expensive to produce and store, carries the risk of viral and prion contamination, and its effect on immune function is unclear [11]. For these reasons, the use of small molecules which are able to target remodeling pathways has been advocated [12]. To this end, peptides such as the gap-junction protein (connexin 43) mimetic (GAP27) [13], the RANK-mimetic peptide [14], the OPG-mimetic peptides [15], the TNF-[alpha] and the RANKL antagonist peptide [16], and a TNF receptor loop peptide mimic [17] have been identified as able to reduce bone resorption and/or promote bone formation.

This work reports the synthesis of a known bone specific cyclic OPG-mimetic peptide (OP3-4) sequence YCEIEFCYLIR, which is based on residues 113–122 of the human OPG sequence [15,18,19], and its complexation with a contrast agent for magnetic resonance imaging (MRI), gadolinium, through a chelating moiety, 1,4,7,10-tetraazacyclododecane-1,4,7,10-tetraacetic acid (DOTA) [20]. The novel peptide formed, DOTA-Gd-OP3-4, was tested for MRI traceability and its direct inhibitory effect on osteoclastogenesis in vitro to offer a novel theranostics agent with the potential to enable the simultaneous diagnosis (or follow up) and treatments of bone affected by metabolic diseases.

## 2. Materials and Methods

### 2.1. Materials

Amino acids protected by 9-fluorenylmethyloxy carbonyl (Fmoc) where purchased from NovaBiochem, Watford, UK. Tenta Gel S NH_2_ resin and Rink amide linker were purchased from Iris Biotech, Marktredwitz, Germany. Dimethylformamide (DMF), dichloromethane, diethyl ether, and methanol were of analytical grade (≥99.5%) and obtained from Fisher Scientific, Loughborough, UK. Triisopropylsilane (TIPS, ≥98.0% GC), phenol (≥99%), trifluoroacetic acid (TFA, ≥99%), and *N*,*N*-diisopropylethylamine (DIPEA, ≥99%) were obtained from Iris Biotech GmbH. 2-(1*H*-benzotriazole-1-yl)-1,1,3,3-tetramethyluronium hexafluorophosphate (HBTU, ≥97%) was purchased from NovaBiochem, UK. Tri-*tert*-butyl 1,4,7,10-tetraazacyclododecane-1,4,7,10-tetraacetate (DOTA-tri-*t*-Bu-ester) was purchased from Sigma Aldrich, Dorset, UK. All other reagents were of analytical grade.

### 2.2. Synthesis of Peptides

The peptides OP3-4 and DOTA tethered OP3-4 (DOTA-OP3-4) were synthesised by solid-phase peptide synthesis (SPPS) using the conventional Fmoc protection/deprotection strategy on Tenta Gel S NH_2_ resin (0.1 mmol equivalent) with DMF as the reaction solvent. The resin was pre-swollen in 7 mL DMF in a 10 mL reaction vessel and the peptides synthesised by first attaching an acid-liable Fmoc-Rink Amide linker to the resin followed by sequential amino acid coupling and deprotection steps as per peptide sequence. HBTU and DIPEA were used for the coupling reaction (×2, 30 min, room temperature) at one and two times the concentration of the amino acids and 20% (*v*/*v*) piperidine in DMF was used for deprotection (2 min, ×3). In all preparations, the resin, linker, and amino acids were added in the molar ratio of 1:4:4, respectively. Each coupling or deprotection step was followed by washing steps (×3 with DMF). After the final deprotection step, the resin was washed with 10 mL DMF (×3), dichloromethane (×4), methanol (×4), and diethylether (×4). The peptides were then dried in a vacuum oven at room temperature until reaching a constant weight (~2 h) and stored at 4 °C for further studies.

**Figure 1:**
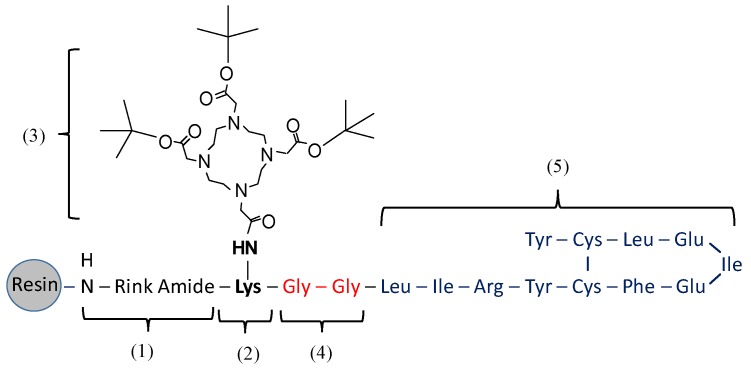
Schematic representation of the assembly of DOTA-OP3-4 by SPPS. Numbered sections indicate the order of assembly. The numbers used refer to: (**1**) the attachment of the rink amide linker; (**2**) the coupling of Fmoc lys (Mtt)–OH; (**3**) the removal of Mtt groups followed by the coupling of DOTA-Tri-t-Bu-ester; (**4**) the coupling of two glycines to provide a spacer; and (**5**) the coupling of the amino acids as per the OP3-4 peptide sequence and subsequent Cys-Cys disulphide bond cyclisation.

In the synthesis of DOTA-OP3-4 (Figure 1), Fmoc-Lys (Mtt)-OH was first coupled to the Rink-amide linker. The Mtt (4-methyltrityl) protecting group was then removed by a series of washes (1 min, ×9) with 1% TFA (*v*/*v*) in DMF until clear; Mtt appears yellow in DMF solution. DOTA-tri-*t*-Bu-ester was then coupled to lysine, followed by the deprotection of the Fmoc protected terminal on lysine. Two glycine amino acids were then sequentially coupled as spacers, followed by the subsequent addition of amino acids as per the OP3-4 sequence (Figure 1).

The peptides were freed from the resin by incubation in a cleavage solution (88% TFA, 5% H_2_O, 5% phenol and 2% TIPS) for 3 h at room temperature. The cleaved peptides were filtered through glass wool, precipitated, and isolated by a series of washing and centrifugation steps in cold diethylether (≤4 °C). The samples were then dried over a stream of nitrogen gas and stored at −20 °C. Crude peptides were dissolved in 0.1% formic acid in methanol at 2 mg/L and characterised on an ion trap mass spectrometer (ITMS) model HTC Plus and a Time-of-Flight mass spectrometer (TOF-MS) model MicrOTOF, both from Bruker Daltonics (Billerica, MA, USA). The systems were optimised for the detection of the peptide *m/z* in every instance and once the peptide was characterised, the systems were re-optimised for the isolation and collection of the purified peptide. Data acquisition was carried out by Compass 1.1, Esquire 5.3, and Hystar 3.1 software (Bruker Daltonics, Billerica, MA, USA).

### 2.3. Cyclisation of OPG Mimetic Peptides

OP3-4 and DOTA-OP3-4 were cyclised by dimethyl sulfoxide (DMSO) oxidation to form cysteine-cysteine disulfide bonds as described in the literature [21], with slight modification. Briefly, peptides (1 mg) were dissolved in 60 mL of oxidising buffer (100 mM NaH_2_PO_4_ and 2 mM Gdn·HCl, 5% DMSO, pH 7.0) and shaken for 12 h. The solution was then acidified with 1 M HCOOH (250 μL) and the peptides purified and analysed by hydrophilic interaction liquid chromatography (HILIC) coupled to ITMS (LC-MS). Cyclisation (disulfide bridge formation) was confirmed by the quantitation of free thiol groups using Ellman’s reagent following kit instructions (Invitrogen Molecular Probes T6060 Thiol and Sulfide Quantitation Kit, from ThermoFisher Scientific, Loughborough, UK) [22].

### 2.4. Gadolinium Chelation into DOTA-OP3-4

Cyclised DOTA-OP3-4 (1 mmoles, in 2 mL ethanol) was mixed with GdCl_3_·6H_2_O (1 mmol) in 5 mL water and the pH adjusted to 6.5 with Na_2_CO_3_ (0.1 mL) and stirred for 15 h at 60 °C. The peptides were then purified by high pressure liquid chromatography (HPLC) using a Solvent A (methanol, 0.1% TFA) and Solvent B (water, 0.1% TFA) gradient from 5% to 60% solvent. The system was coupled to the non-destructive soft-ionisation electrospray ITMS.

### 2.5. Chromatographic Separation of the Peptides by LC-MS

The peptide synthesis products were separated by HILIC on an HP 1200 HPLC (Agilent Technologies, Santa Clara, CA, USA) equipped with a TSK-Gel Amide 80 HILIC column (250 mm (length) × 4.6 mm internal diameter and 5 μm particle size) from TOSOH Bioscience (Tokyo, Japan). The system used a binary mobile phase of solvent A (acetonitrile, 0.1% (*v*/*v*) formic acid) and solvent B (water, 0.1% (*v*/*v*) formic acid) at a flow rate of 300 µL/min. The gradient elution program was: 0–50% solvent B for 0–20 min for OP3-4 and 5–90% solvent B for 0–45 min for both DOTA-OP3-4 and DOTA-Gd-OP3-4. The peptides were dissolved in 0.1% (*v*/*v*) formic acid in methanol at 20 µg/g and the injection volume was 100 μL. The tube from the column was divided into two channels of equal length and diameter, with one leading to the detector (ITMS) while the other led to the sample-collecting vessel where purified peptides were collected at the point of detection. The purified peptides were lyophilized and stored at −20 °C.

### 2.6. Tandem MS

ITMS was first optimised to detect the putative ions from the SPPS products in the full scan mode, followed by their isolation in the single ion monitoring mode (SIM). Energy was then applied to the system to fragment the isolated putative ion (MS/MS or MS2) and the resulting fragmentation products were scanned in product ion mode. Depending on the intensity and amount of the fragmentation products from MS/MS, the most intense fragment (base peak) was isolated and fragmented further (MS3), which made further confirmation of the identity of the peptide possible. The process was repeated with subsequent base peaks until the obtained peaks were within the noise signal.

### 2.7. MRI Analysis

The peptides (DOTA-OP3-4 and DOTA-Gd-OP3-4) were first dissolved in a minimum volume of DMSO and then diluted to a desired peptide concentration (0–10 µg/mL and 0.5% by volume DMSO) in phosphate-buffered saline (PBS) and the pH adjusted to 7.2 using 0.1 M HCl. DOTA-Gd and DOTA-OP3-4 were used at 10 µg/mL as the positive and negative controls, respectively. The DOTA-Gd-OP3-4 sample was then double diluted ten times from 10 µg/mL to 0.005 µg/mL and each sample (1 mL) was placed into different wells of a 24-well tissue culture plate for MRI analysis. The analysis was performed in T1 weighted scan mode using the Siemens AVANTO 1.5T MRI scanner with parameters set as: echo time (TE) 13, repetition time (TR) 200, and SL 1 mm. The analysis was conducted at the Clinical Imaging Science Centre, Brighton and Sussex Medical School, Brighton, UK.

### 2.8. Cell Viability Studies

Osteoblastic cells, the SaOS-2 cell line (ATCC, Manassas, VA, USA), were cultured to 85% confluence in McCoy’s 5A culture medium (without l-glutamine) supplemented with 10% foetal bovine serum (PAA Laboratories GmbH, Cölbe, Germany). The cells were then trypsinised (5 mL trypsin, 37 °C for 6 min), washed, and seeded into 96-well plates at a 10^4^ cells/well seeding density. The cells were then treated with different peptide concentrations (0–200 µM) and incubated (37 °C, 5% CO_2_) for 24 h, after which the cell viability and cytotoxicity were evaluated.

Cell viability was studied by assessing the ability of the cells to metabolically reduce a tetrazolium salt 3-(4,5-dimethylthiazol-2-yl)-5-(3-carboxymethoxyphenyl)-2-(4-sulfophenyl)-2*H*-tetrazolium (MTS) to a soluble formazan derivative following kit instructions (CellTiter 96^®^ AQueous One Solution Cell Proliferation Assay from Promega, Southampton, UK), with slight modification. Briefly, cells in a 96 well plate were washed three times with PBS and re-supplemented with 100 µL of culture medium per well. MTS/PMS (20 µL) solution (1:20 *v*/*v*) was then added to each well and the cells incubated for 2 h. Control cells were not exposed to peptides. The amount of formazan produced (purple colour) was measured at an absorbance wavelength of 490 nm using the Biochrom Asys UVM 340 plate reader equipped with Micro Win 2000 software (Biochrom Ltd., Cambridge, UK).

Cytotoxicity of the peptides was studied by assessing the amount of lactate dehydrogenase (LDH) released from cells as a measure of the number of lysed cells following kit instructions (CytoTox 96^®^ Non-Radioactive Cytotoxicity Assay, Promega, UK). Briefly, after peptide treatment, cell culture medium isolated from every well was centrifuged (2000 rpm, 5 min) and the debris-free supernatant transferred to a clean 90 well plate. CytoTox 96^®^ Reagent (100 µL) was added to each well and incubated for 30 min. The kit stop solution was added and absorbance measured at 490 nm. Positive control cells were completely lysed with Triton-X before centrifugation to estimate the maximum toxicity value in the same culture conditions measured as total LDH into the tissue culture supernatant. Negative control cells were not lysed. All control cells were not exposed to peptides.

Epi-fluorescence microscopy analysis for viable cells in cultures treated with 100 µM of each peptide was performed after Hoechst 33,258/Propidium iodide (H/PI, from Invitrogen, Carlsbad, CA, USA) nuclear staining (50 ng/mL) at an H:PI ratio of 1:1 per well in a 24 well culture plate. Diffusely H positive nuclei (blue and live) and diffusely PI positive nuclei (pink and dead) were counted and the number of live cells was presented as a percentage of the total number of cells per field. At least 200 cells from different randomly picked areas were counted per well.

### 2.9. Monocyte Isolation and Osteoclastogenesis Studies

Ethical approval for human blood studies was acquired from the University of Brighton Faculty Research Ethics and Governance Committee, Brighton, UK. Human whole blood (6 mL) from healthy consented volunteers was layered on Histopaque (3 mL, density: 1.077 g/mL, from Sigma Aldrich, UK) in heparinised tubes (9 mL Vacuette NH Sodium Heparin, Greiner Bio-One International GmbH) and centrifuged (450× *g*, 30 min). The monocyte-rich buffy coat layer was isolated by gentle pipette aspiration and washed in 8 mL PBS (×3) by centrifugation (250× *g*, 15 min) to remove the platelet-rich plasma fraction. The cells were then resuspended in 1 mL αMEM (PAA Laboratories GmbH, Germany) medium and seeded into 24 well plates at a 5 × 10^5^ cells/well seeding density. After a 3-h incubation (37 °C, 5% CO_2_), non-adherent cells were rinsed with PBS (×3) and the adherent cells cultured for 24 h in αMEM medium supplemented with 10% (*v*/*v*) FBS and Penicillin/Streptomycin (1×).

Monocyte differentiation into osteoclasts was induced by spiking the cells with human recombinant RANKL (rh RANKL) and Macrophage Colony Stimulating Factor (rh M-CSF), both purchased from Invitrogen, UK. Firstly, monocytes were treated with different concentrations of rh RANKL (0, 10, 50, and 100 ng/mL) in the presence of 25 ng/mL rh M-CSF and over different time periods (two, four, and six days). Osteoclastogenesis inhibition was then studied by culturing monocytes in αMEM medium supplemented with 10% (*v*/*v*) FBS, Penicillin/Streptomycin (1×), 50 ng/mL rh RANKL, and 25 ng/mL rh M-CSF for four days in the presence or absence of 50 µM peptides (OP3-4, DOTA-OP3-4 and DOTA-Gd-OP3-4). The positive control was treated with rh OPG (50 ng/mL) and the negative control had no treatment. The degree of osteoclastogenesis was assessed by tartrate-resistant acid phosphatase (TRAP) staining using a commercial kit (387A for cytochemical staining from Sigma Aldrich, UK) according to the manufacturer’s instructions. TRAP positive multinucleated cells were counted using a Nikon Eclipse T*i*-U microscope equipped with a Nikon DIGITAL SIGHT DS-Fi1 camera (Nikon Corporation., Tokyo, Japan) and NIS-Elements BR 3.2 software (Nikon Corporation., Tokyo, Japan) was used to count the cells in six different randomly selected areas in each well.

## 3. Statistical Analysis

All statistical analyses were performed by using analysis of variance (ANOVA) (software: Minitab Version 15, Minitab Inc, State College, PA, USA). Whenever ANOVA indicated the groups were significantly different, a *t*-test for independent samples was performed. Samples were considered significantly different at *p* ≤ 0.05.

## 4. Results and Discussion

### 4.1. Peptide Synthesis and Characterisation

Due to the lack of peptide standards for comparative verification of the successful synthesis of the putative peptides, two mass spectrometry analysis approaches were used: (1) TOF-MS, to benefit from its high mass resolution and accuracy (up to five decimal places), high linearity, and wide scan range (50 to 3000 *m/z*); and (2) ITMS, to benefit from its high sensitivity and capacity to sequentially trap and fragment desired ions so as to identify the building units of a molecule that would confirm successful synthesis.

The MS acquisition for the putative peptides was first performed in full scan mode using TOF-MS, providing an overview of the products. The theoretical *m/z* of the peptides and their respective fragment ions were generated using the MS-product tool of the online program ProteinProspector V5.10.0. [23]. Successful cyclisation through the cysteine-cysteine disulfide bridge formation was confirmed by the absence (95% reduction) of free thiol groups in both OP3-4 and OP3-4-DOTA after the oxidation reaction (data not shown).

**Figure 2:**
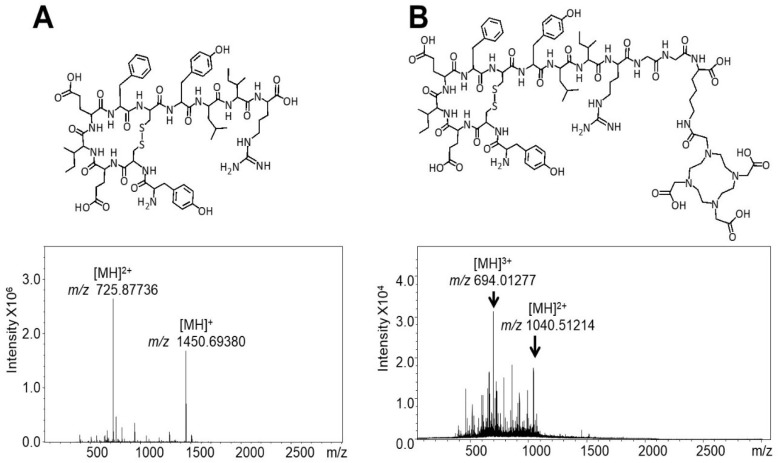
TOF-MS spectra and Structure of the peptides: (**A**) OP3-4 and (**B**) DOTA-OP3-4.

The molecular structure and mass spectra of the OP3-4 peptide (*m/z* 1450.7) synthesis products acquired by TOF-MS are shown in Figure 2A, where the putative ions [M + 2H]^2+^ (*m/z* 725.87, base peak) and [M + H]^+^ (*m/z* 1450.68) are annotated. Although incomplete fragments were seen in the spectra of OP3-4, the fragments were not sufficiently abundant to allow their conclusive identification from the background noise. The analysis of the synthesis products of the novel DOTA-OP3-4 peptide by TOF-MS showed numerous undesired product ions. As a result, ITMS, with its ability to trap specific desired ions using the single ion mode (ITMS-SIM), was used to confirm the presence of the putative ion. The full scan MS spectrum of the synthesis products of the novel DOTA-OP3-4 peptide (*m/z* 2080.2) showed ions with *m/z* 694.1 (base ion) and *m/z* 1040.5, which correspond to the putative peptide ions [M + 3H]^3+^ and [M + 2H]^2+^, respectively (Figure 2B). The introduction of a lysine-glycine-glycine spacer between the OP3-4 sequence and the DOTA moiety in DOTA-OP3-4 was essential to avoiding potential steric hindrance during the synthesis and to maximise the exposure of the peptide to the relative cell receptor. The numerous product ions observed in the MS spectra of DOTA-OP3-4 may be the effect of using 0.1% (*v*/*v*) TFA in the presence of the cysteine-rich peptide during the additional step to remove lysine protecting groups (Mtt) and the subsequent coupling of DOTA. The interaction of both the terminal carboxyl groups on the growing peptides and of the DOTA moiety with the side chain groups on the peptide during the synthesis and the cleavage steps may also have led to intra- and intermolecular interactions, resulting in numerous incomplete and undesired peptidic chains.

**Figure 3:**
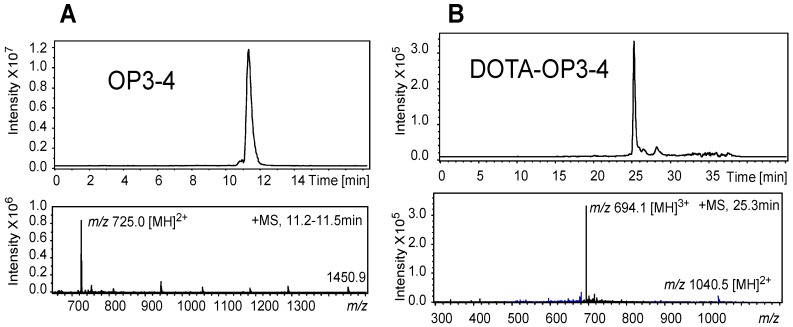
LC-MS chromatographic separation profile of the putative peptides (top) (**A**) OP3-4 (*m/z* 1450.9) and (**B**) DOTA-OP3-4, (*m/z* 2081.4) with their respective ITMS ion mass spectra (bottom) eluted as peaks at 11.2–11.5 and at 25.3 min. The purity of the putative peptides, estimated by integration of the peak areas, was >95% (OP3-4) and 90% (DOTA-OP3-4).

The analysis of the OP3-4 peptide by LC-MS showed the peptide to account for 80% of the crude synthesis products and a purity of >98% could be achieved after LC-MS purification with ITMS as the detector (Figure 3A). Despite the presence of numerous intense peaks corresponding to undesired ions, the ion corresponding to DOTA-OP3-4 was found to account for 60% of the crude synthesis products by LC-MS. This disparity in the intensity of the synthesis products by TOF-MS and LC-MS coupled to ITMS could be due to differences in the volatility of the ions in the MS [24] as smaller molecules are more volatile than larger ones in MS. Although TOF-MS gives highly accurate *m/z* for the ions, the system is not as sensitive as the ITMS. Since ITMS allows for optimisation of the detection of the desired ions within a specified *m/z* range, and LC-MS separates molecules according to their polarity, a more accurate result of the purity and/or yield of the synthesis could be obtained. Indeed, after isolation of the putative ion, DOTA-OP3-4 was found to account for >95% of the isolated product (Figure 3B).

**Figure 4:**
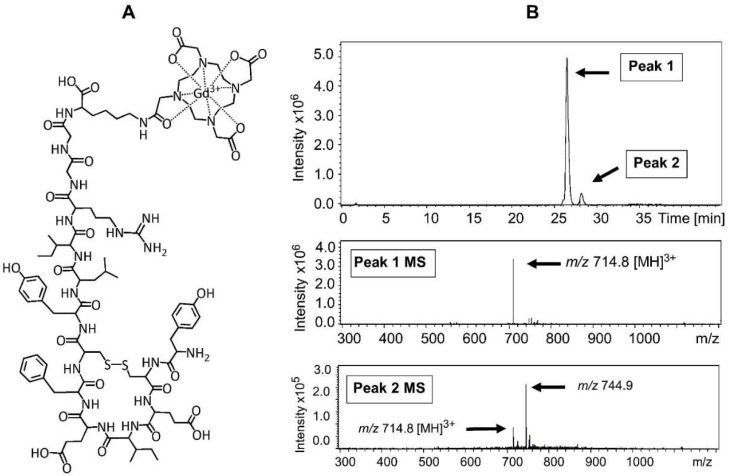
(**A**) Schematic representation of DOTA-OP3-4 with Gd^3+^ conjugate (DOTA-Gd-OP3-4); (**B**) Chromatographic separation profile of the purified DOTA-Gd-OP3-4. The ITMS spectra of the ions eluted in peak 1 (putative peptide) and peak 2 (contaminating fraction) are also shown (bottom). The purity of the peptide, estimated by integration of the peak areas, was >90%.

Successful chelation of the gadolinium ion (Gd^3+^) onto purified DOTA-OP3-4 to produce DOTA-Gd-OP3-4 (Figure 4A) was confirmed by LC-MS, where the peak with *m/z* 694.1 for DOTA-OP3-4 peptide shown in Figure 3B was replaced by a base peak with *m/z* 714.8, corresponding to the ion [M + 3H]^3+^ of DOTA-Gd-OP3-4 after the chelation of Gd^3+^ (Figure 4B). LC-MS allowed the isolation of the peptide from salts, non-chelated Gd^3+^, and other synthesis products, and the analysis of the purified DOTA-Gd-OP3-4 sample showed no evidence of residual free Gd^3+^ ions.

Gadolinium is a lanthanide metal commonly used in clinics as an MRI contrast agent. Gadolinium is used in its ionic form chelated by large, non-toxic linear or macrocyclic organic molecules that form stable and biochemically inert gadolinium contrast agents (GCA). This sequestration of Gd^3+^ with organic chelates circumvents the known toxicity of free Gd^3+^ in humans and ensures their excretions from the body through kidney clearance. However, these GCA, in particular those of linear chelates such as Omniscan (gadodiamide), Magnevist (gadopentetate dimeglumine), and Optimark (gadoversetamide), are associated with slow excretion rates due to accumulation within the body (i.e., in the liver) and toxicity from free Gd^3+^ ion release due to transmetallisation with Zn^2+^, Cu^2+^, and Ca^2+^ in vivo [25,26]. Macrocyclic GCAs such as dotarem (Guerbet) and gadovist (Bayer Schering Pharma AG) are less toxic as they bind Gd^3+^ more tightly and are more stable with low dissociation rates than linear chelates. However, these GCAs, like linear ones, are associated with short circulation in the body and inefficient discrimination between diseased and normal tissue seen in use of low molecular diethylenetriaminepentaacetic (DTPAs) [25,26].

Through this work, tissue-specific imaging may be improved by the incorporation of a peptide sequence specific for the cross talk of bone cells, the RANK-RANKL pathway, and the potential toxicity of Gd^3+^ is circumvented by the use of DOTA, a macrocyclic chelate for Gd^3+^ [27]. Indeed, some DOTA-based Gd contrast agents are FDA approved and are among the most widely used contrast agents in clinical imaging with well-established safety and MRI efficacy [27,28].

In the chelation of Gd^3+^ into DOTA-OP3-4, the DOTA moiety acts as a polydentate ligand and envelops the metal cations, in this case complexing Gd^3+^, to give an MRI visible peptide. The coordination of the DOTA ligands and metal ion in the complex depends on the conformation of the ligand and geometric tendencies of the metal cation [29,30]. On its own, DOTA acts as an octadentate ligand, binding the metal through four amino and four carboxylate groups. In this study, the DOTA molecule acts as a septadentate since one of the carboxylate groups is used in the formation of a covalent bond with the peptide. However, a carboxylate group from the amino acid linking DOTA and the peptide provides the eighth ligand and restores the octadentate state, forming a highly stable coordination complex [30].

**Figure 5:**
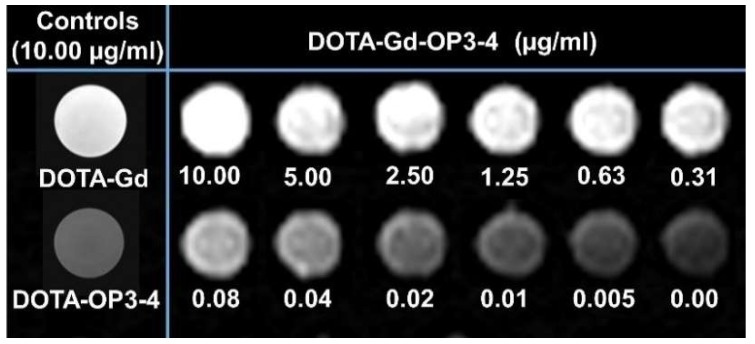
T1 weighted MRI scan of DOTA-Gd-OP3-4 showing a concentration-dependent signal intensity. The peptides, pre-dissolved in DMSO, were diluted to the desired concentration in PBS buffer (1 mL) and the MRI signal acquired at the same time using the parameters: SL5, TE 8.7, and TR550. DOTA-Gd (MW 557.64 g/mol) and DOTA-Gd-OP3-4 (MW 2141.4 g/mol) were prepared at a concentration of 1 µg/ml, corresponding to 1.79 µM and 0.47 µM Gd, respectively.

Analysis of the result obtained from a T1 weighted MRI scan confirmed the successful chelation of Gd^3+^, with DOTA-Gd-OP3-4 producing a brighter signal than DOTA-OP3-4, which had a signal similar to the sample with PBS solutions only (Figure 5). Moreover, the intensity of the T1 signal from DOTA-Gd-OP3-4 samples was directly proportional to the concentration of the peptide.

The coordination of the DOTA ligands and metal ion in the complex depends on the conformation of the ligand and geometric tendencies of the metal cation [29,30]. On its own, DOTA acts as an octadentate ligand, binding the metal through four amino and four carboxylate groups. In this study, the DOTA molecule acts as a septadentate since one of the carboxylate groups is used in the formation of a covalent bond with the peptide. However, a carboxylate group from the amino acid linking DOTA and the peptide provides the eighth ligand and restores the octadentate state, forming a highly stable coordination complex [30].

Although the potential theranostic benefits of the developed peptide are clear, the minimum peptide concentration that would be required for a visible change in the MRI signal intensity in vivo remains to be determined through future relaxivity studies [31,32]. Indeed, relaxivity calculation requires the different concentrations of the peptide to be analysed separately in T1 and T2 modes and using the respective r1 and r2 to determine relaxivity [31,32,33]. Although the in vivo relaxivity and safety of the DOTA-Gd moiety are well-established, [28,32,34], the new peptide complex presents a different immediate molecular environment that can affect the MRI profile of Gd^3+^ [35,36]. The importance of relaxivity studies in vivo is supported by the effect that the molecular environment has on the MRI profile, leading investigators to question the clinical efficacy of T1 relaxivity measurements performed in vitro in simple media and temperatures different from those of living tissue [32,33,34,36].

In addition, although TOF-MS and LC-MS demonstrated the successful synthesis of peptides, it was necessary to perform tandem MS for additional confirmation of the results due to the lack of standards. The ions to be fragmented were acquired in product ion scan mode, isolated, and further fragmented to produce smaller units that allowed peptide identification and the confirmation of successful synthesis. In all cases, the voltages for the optimal transfer of the ions from the LC into the ion trap, their stabilisation in the trap, and fragmentation were optimised. A list of the peptides, isolated ions thereof (SIM), and fragmentation products are given in Table 1.

### 4.2. The Effect of the Peptides on Cell Viability and Osteoclastogenesis

**Figure 6:**
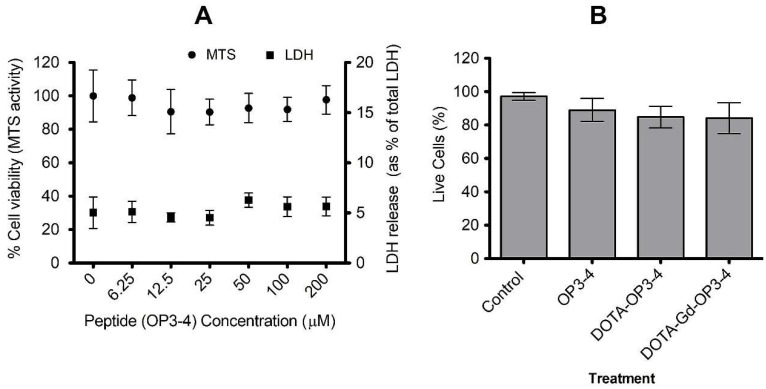
OP3-4, DOTA-OP3-4, and DOTA-Gd-OP3-4 have no significant effect on the viability of SAOS-2 cells. (**A**) The cells were cultured with different concentrations of OP3-4 peptide for 24 h and the percentage of dead and live cells determined by assaying for LDH in supernatant and MTS assay, respectively. Absorbance was measured at 490 nm. (**B**) The effect of derivatised OP3-4 peptides (at 100 µM) on cell viability was determined by fluorescence microscopy after H/PI live and dead cell staining (**B**). Data is mean ± SE, *n* = 6.

The effects that the peptides have on cells was first studied by spiking SAOS-2 cells with different concentrations of OP3-4. MTS and LDH assay results showed cell viability within a wide range of peptide concentrations (Figure 6A). The H/PI study of the effect of OP3-4-DOTA and OP3-4-DOTA-Gd on the SAOS-2 cells relative to OP3-4 at a fixed concentration (100 µM) showed no significant effect on cell viability (Figure 6B). Although there appears to be a decrease in cell viability from the control to OP-3-4 to DOTA-OP3-4 and DOTA-Gd-OP3-4, the differences were not significant to the control and across the different treatment groups (*p* > 0.05).

### 4.3. Osteoclastogenesis Inhibition Studies

**Figure 7:**
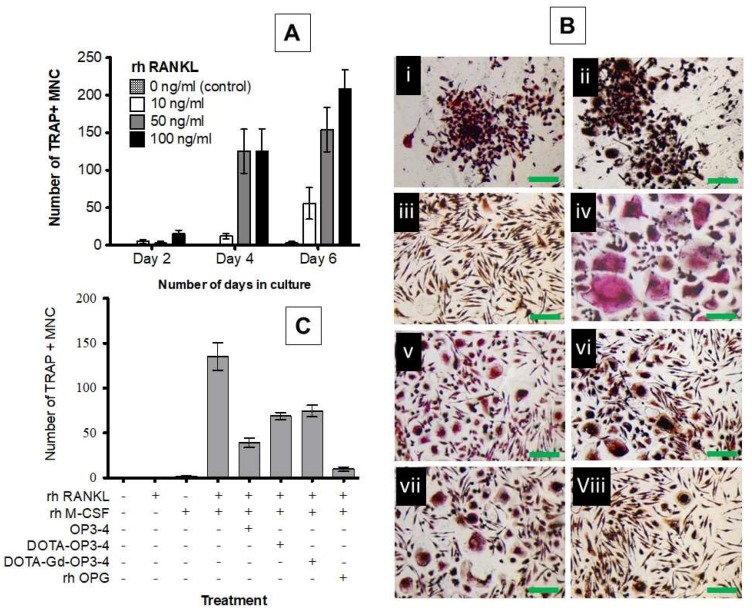
(**A**) Graphical representation of the effect of rh RANKL concentration and time in culture on osteoclastogenesis. (**B**) The effect of peptides on osteoclastogenesis after a four-day culture with (**i**) no cytokines or peptides, (**ii**) rh RANKL only, (**iii**) rh M-CSF only, (**iv**) rh RANKL and rh M-CSF, (**v**) OP3-4, (**vi**) DOTA-OP3-4, (**vii**) DOTA-Gd-OP3-4, and (**viii**) rh OPG (positive control): iv–ix had rh RANKL (50 ng/mL) and rh M-CSF (25 ng/mL). (**C**) Graphical representation of osteoclastogenesis inhibition by OPG mimetic peptides.

The studies conducted to assess the effect of cytokine (rh RANKL and rh M-CSF) concentration over two, four, and six days in culture showed that the amount of TRAP-positive giant multinucleated cells (MNC) per well increased with the increase of both cytokine (rh RANKL) concentration and culturing time (Figure 7A). The number of TRAP-positive MNCs increased significantly after four days in cultures treated with 50 ng/mL and 100 ng/mL rh RANKL. However, osteoclastogenesis in cultures treated with 50 ng/mL rh RANKL was not significantly different from 100 ng/mL at day 4 and 6 (*p* > 0.05). As such, the rh RANKL concentration of 50 ng/mL and a culture time-period of four days were adopted for studies on osteoclastogenesis inhibition by the peptides.

Representative images of the cells after four days of culture in the presence or absence of the cytokines and in the presence or absence of the peptides are given in Figure 7B. The cells that did not receive cytokines and those treated with rh RANKL only were TRAP negative, morphologically small, and round, with a tendency to form clusters (Figure 7B(i and ii)). However, the fact that this clustering phenomenon was observed in cells that did not receive either cytokine suggests that it could be due to other factors apart from rh M-CSF and/or RANKL and more studies are necessary to clarify that. On the other hand, the cells treated with only rh M-CSF were TRAP negative and morphologically narrow and stretched (Figure 7B(iii)), while those treated with both cytokines differentiated into cells that were much larger (>×10), multinucleated, morphologically irregular, and TRAP positive (Figure 7B(iv)). This is consistent with the established knowledge that M-CSF and RANKL induce the migration of monocytes towards each other, resulting in cell fusion to produce a giant osteoclast like TRAP-positive MNC [37,38,39]. The size and amount of TRAP-positive MNCs was seen to reduce in cultures treated with the peptides (Figure 7B(v–vii)) and in the positive control (rh OPG) (Figure 7B(viii)). This result was also quantitatively confirmed by counting the number of TRAP-positive cells with each respective treatment (Figure 7C). The observed potency of OP3-4 in osteoclastogenesis inhibition is consistent with previous findings [17,18] and was significantly higher than that observed for DOTA-OP3-4 and DOTA-Gd-OP3-4. However, all the peptides significantly reduced the RANKL/M-CSF-induced osteoclastogenesis.

Indeed, the presentation and binding of the developed OPG mimetic peptides to RANK may have been specific and sufficient to inhibit RANK-RANKL interaction. It is well established that although the molecules of the TNF superfamily (TNFSF), to which RANK and RANKL belong, are similar in structure, several members of this cytokine family show significant sequence diversity [40]. Consistent with this observation, studies on the 3D structures and mechanism of members of the TNFSF such as TNFα [41], TNF-β [42], RANKL/RANK [43,44], sTNF-R1[45], CD40L [46], TRAIL-DR5 [47], and many more, have revealed that these cytokines recognise their ligands or receptor with specificity and in most cases, exclusively [40,44]. In the present study, the potency of the rh OPG was significantly higher than that of the peptides although this could be, in part, due to the fact that OPG interacts with RANKL through multiple active sights and domains when compared to the peptides. However, the observed potency of the OPG mimetic peptides, which are smaller molecules compared to the OPG ligand, may indicate a higher degree of specificity that is enough for a desired effect of reducing osteoclastogenesis. It is also worth noting that the dosage used for peptides and OPG was not equimolar. Therefore, the data were not directly comparable due to the differences in the number of active domains and molecular weight of the natural protein and of the synthetic peptides. As such, the cultures treated with OPG should only be considered as a positive control.

The peptide is an OPG mimic designed to bind to RANKL and attenuate osteoclastogenesis. This in vitro cell work demonstrated the theranostics potential of the peptide, DOTA-Gd-OP3-4, in future clinical applications. However, in vivo evaluations of the peptide biodistribution, tissue retention time, and bone targeting properties are necessary to inform on the safety and potential clinical efficacy of the developed theranostics.

## 5. Conclusions

OPG mimetic peptide (OP3-4) and its novel derivatives DOTA-OP3-4 and MRI detectable DOTA-Gd-OP3-4 were successfully synthesised with DOTA-Gd-OP3-4, showing a concentration-dependent MRI T1 signal. The OP3-4 was found to be noncytotoxic on osteoblasts at the concentrations examined and its derivatisation into DOTA-OP3-4 and DOTA-Gd-OP3-4 did not reduce cell viability. Moreover, their potency in the inhibition of RANKL/M-CSF-induced osteoclastogenesis in monocytes was clearly proven. Therefore, this work provides evidence in support of a novel theranostics tool for a more efficient and specific treatment of metabolic bone diseases, such as osteoporosis and metastases.

## Figures and Tables

**Table 1:** Tandem MS of OP3-4 and DOTA-OP3-4 peptides using ITMS.

	TOF-MS (Full Scan MS)		ITMS MS-MS Product Ions (Ion Trap)			
Peptide	(*m/z*)	Tentative Assignment	SIM (*m/z*)	MS/MS Product Ion (*m/z*)	Tentative Assignment	MS/MS Scan Range
**OP3-4**	1451.6960	[M + H]^+^				500–1500
	726.377808	[M + 2H]^2+^	726	886	b_7_-2H	300–1200
				733	b_6_-H_2_O	
				715	b_6_-2H_2_O	
				564	y_4_	
**DOTA-OP3-4**	1040.52070	[M + 2H]^2+^				
	694.01938	[M + 3H]^3+^	694	987		300–1200
				691	b_11_-2H_2_O-NH_3_^2+^	
				596		
				514	y_7_^2+^	
				458	y_6_^2+^	
				401	y_5_^2+^	
